# Undifferentiated melanoma: a molecular study of a fatal metastatic “atypical fibroxanthoma (AFX)”

**DOI:** 10.1007/s00428-025-04265-5

**Published:** 2025-10-11

**Authors:** Simon Haefliger, Baptiste Ameline, Veronika Blum, Matthias S. Matter, Beata Bode-Lesniewska

**Affiliations:** 1 Department of Quantitative Biomedicine, University of Zurich, Zurich, Switzerland; 2 Institute for Molecular Health Sciences, ETH Zurich, Zurich, Switzerland; 3 Life Science Zurich Graduate School, ETH, University of Zurich, Zurich, Switzerland; 4 Pathology, Institute of Medical Genetics and Pathology, University Hospital of Basel, Basel, Switzerland; 5 Department of Oncology, Cantonal Hospital Lucerne, Lucerne, Switzerland; 6 Institute of Pathology, Cantonal Hospital Lucerne, University of Zürich, Spitalstrasse, CH-6000 Lucerne, Switzerland

**Keywords:** Undifferentiated melanoma, Atypical fibroxanthoma, Next-generation sequencing, Methylomic analysis

## Abstract

Diagnosing undifferentiated spindle cell and pleomorphic tumours of the sun-exposed skin of elderly patients is common and challenging. This paper presents the case of a 64-year-old man with a tumour that was initially diagnosed as an atypical fibroxanthoma, but which metastasised to the bone and lung, resulting in death within two years. Extensive comparative molecular studies were performed using next-generation sequencing (NGS) and methylomic analysis, which demonstrated that the skin tumour and the bone metastases corresponded to the same neoplasms. In addition to other aberrations, NGS analysis of both tumour manifestations revealed *NF1* gene mutations, suggesting a diagnosis of undifferentiated melanoma. Interestingly, however, the methylomic analysis grouped the tumours with the “atypical fibroxanthoma/pleomorphic dermal sarcoma (AFX/PDS)” class of the reference cases, rather than with conventional or desmoplastic melanomas. Molecular studies may help to reveal the genetic basis of difficult-to-classify undifferentiated skin neoplasms as well as help to estimate their biological potential.

**Supplementary Information:**

The online version contains supplementary material available at 10.1007/s00428-025-04265-5.

## Introduction

Despite its wide phenotypic diversity and several subtypes, malignant melanoma (MM) typically exhibits characteristic microscopic and immunohistochemical properties, leading to straightforward histopathological diagnoses [4]. In rare cases, partial or complete loss of expression of proteins typical of melanoma can make routine diagnosis challenging [2, 14, 15]. Dedifferentiated malignant melanoma (DMM) shows an abrupt transition from typical MM to a malignancy that lacks melanoma properties and/or shows heterologous features. This was previously occasionally interpreted as a ‘collision’ tumour of two independent neoplasms. However, molecular studies have demonstrated close genetic similarities between the two components in such cases, confirming the hypothesis of dedifferentiation in MM. Even rarer are undifferentiated melanomas (UMM), where no residues of typical MM are seen, even in extensively studied tumours. Such cases may evade routine histopathological diagnosis and are subject to pitfalls.

Diagnosing primary cutaneous tumours in chronically photodamaged skin that lacks any signs of specific differentiation is a common and notoriously difficult problem in dermatopathological samples. Following extensive immunohistochemical studies, a diagnosis of atypical fibroxanthoma (AFX) or pleomorphic dermal sarcoma (PDS) can be made by exclusion. The diagnosis of AFX is prognostically favourable when the tumour is strictly confined to the cutis, while PDS is diagnosed when there is any infiltration of the subcutaneous tissue or beyond. This carries a less favourable prognosis and a risk of local recurrence or lymph node metastasis [1, 6, 12]. At the molecular level, both AFXs and PDSs carry a similar actinic imprint with a UV radiation signature [8], that is related to that of cutaneous squamous cell carcinoma [3]. Similar DNA methylation profiles for both lesions are typically observed, alongside a high mutational load and mutations of the *TP53*, *NOTCH1*, *FAT1* and *TERT* promoter genes, as well as alterations in the *CDKN2A* and *CDKN2B* genes on NGS. Over the last few decades, substantial knowledge has been accumulated on the genetic background of malignant melanomas [5]. The pathogenesis of MM variants involves various genetic alterations that disrupt some key signalling pathways, such as the MAPK pathway. The detection of the pathogenic *BRAF* V600E gene mutation in malignant melanoma is relevant both for diagnosis and treatment and can nowadays be easily tested using immunohistochemistry. Other typical alternative mutations affect the *TP53*, *NF1*, *NRAS* and the *TERT* promoter genes. The molecular signature of a MM detected in a malignant skin neoplasm or metastatic tumour suggests a melanocytic line of differentiation, even if the typical immunoprofile is lacking [2, 7, 14, 15].

We describe a well-documented case of a cutaneous tumour in a 64-year-old man, initially diagnosed as an AFX, with fatal haematogenous metastatic disease within several months and provide comparative molecular data of the primary and metastatic tumour tissues.

## Case description, morphological and immunohistochemical analysis

**Fig. 1:**
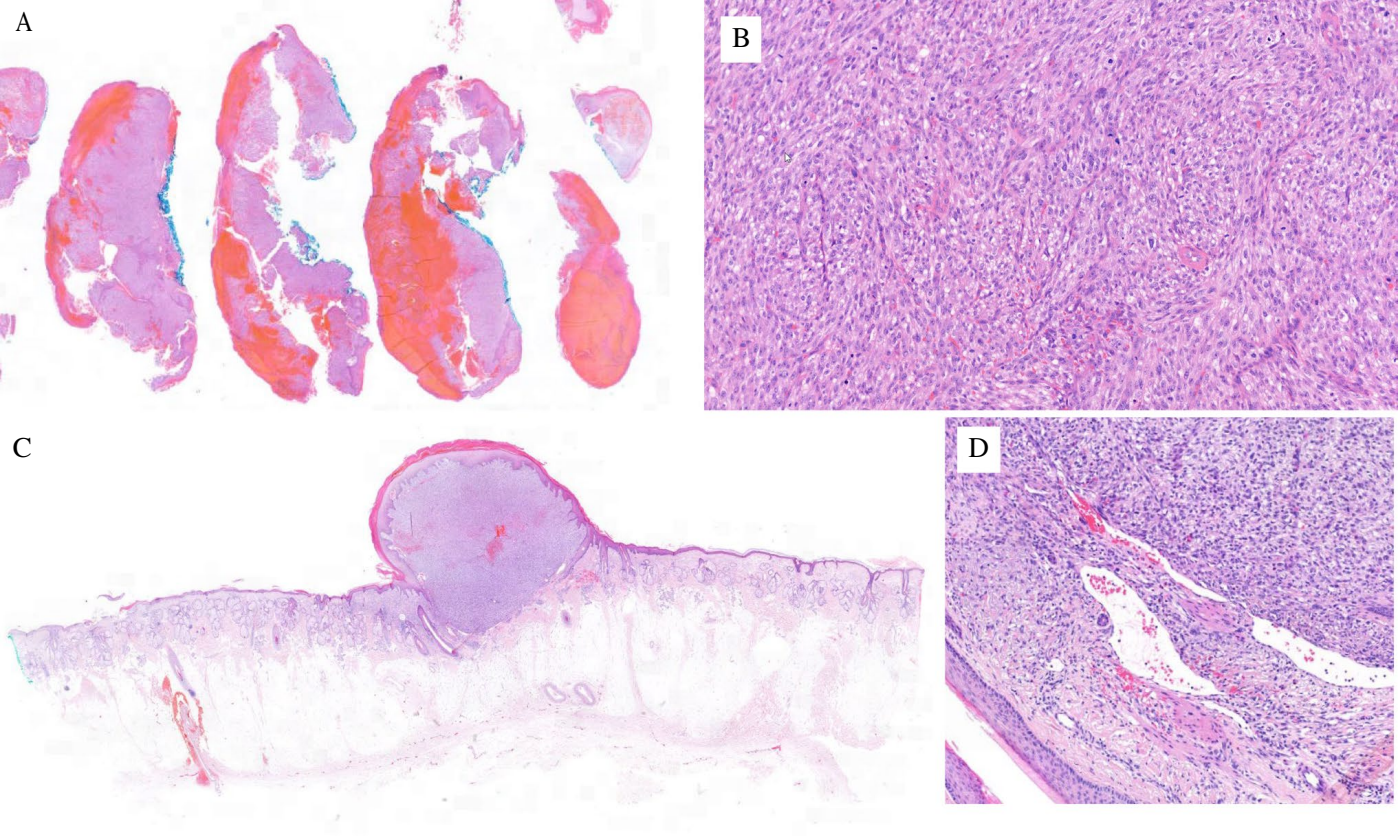
Histology of the primary skin tumour: (**A**) Shave biopsy of an ulcerated and bleeding nodule on the head. (**B**) Spindle cell and pleomorphic undifferentiated neoplasia, immunohistochemically (not shown) completely negative for epithelial, melanocytic, myogenic and endothelial markers. (**C**) Complete re-resection of an exclusively intradermally located tumour. (**D**) Close relation of the tumour tissue to the vascular spaces at the periphery of the tumour

**Fig. 2:**
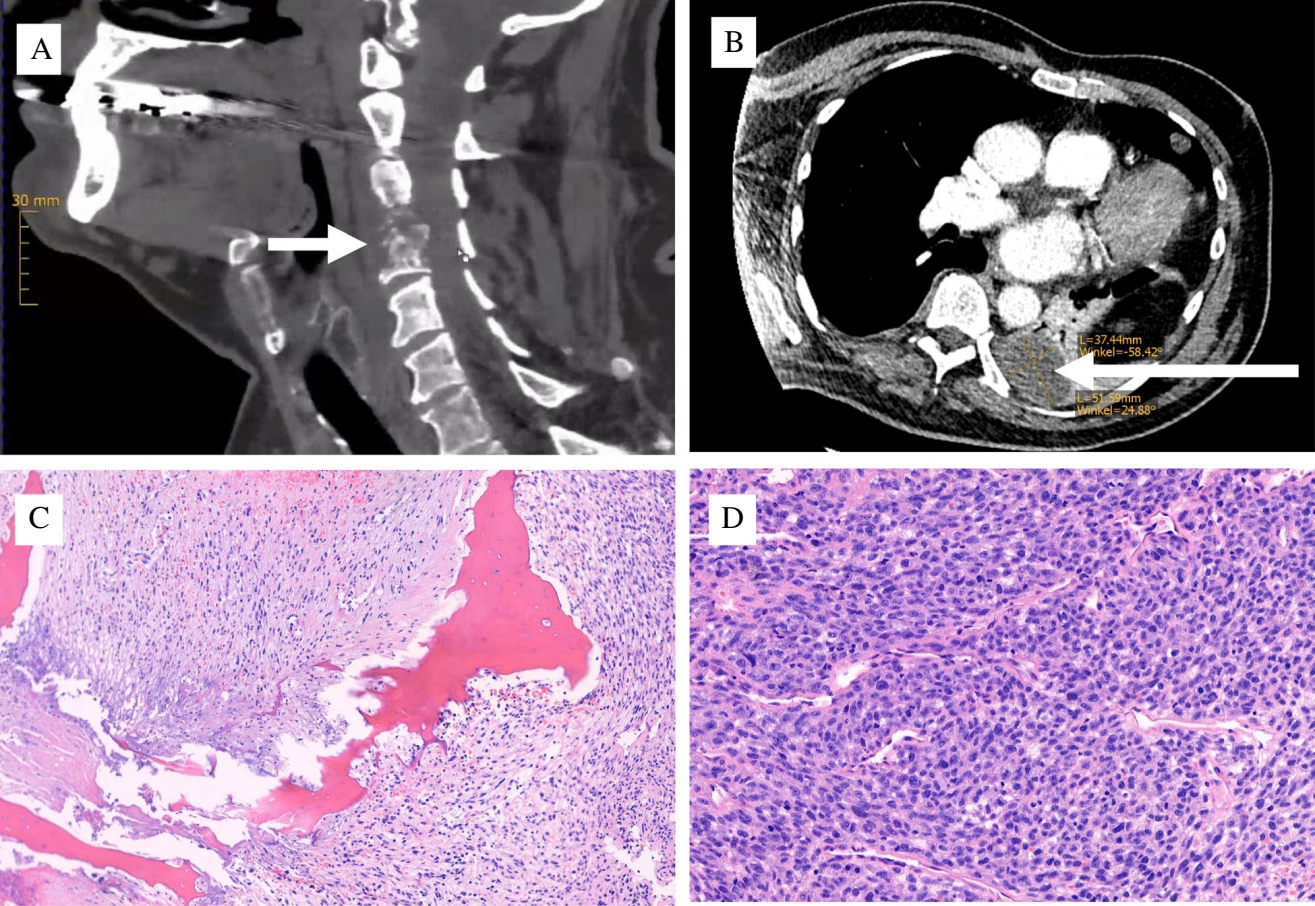
Metastatic disease 16 months following the resection of the skin tumour: (**A**) CT of the destructive bone metastasis of the cervical spine (arrow). (**B**) Lung metastasis (arrow) (CT). (**C**) Bone destructive and extensively necrotic tissue in the surgical decompression specimen from the cervical spine. (**D**) In the viable areas of undifferentiated pleomorphic, well vascularised tumour, there was no immunohistochemical expression of line specific markers (not shown)

A 64-years-old male patient with no relevant past medical history (especially no history of malignant tumours) noticed a growing and bleeding nodular skin lesion measuring 2 cm on his head. A shave biopsy performed in an external dermatology practice (Fig. 1A–B) revealed a spindle and pleomorphic cell proliferation that did not exhibit any morphological or immunohistochemical signs of specific line of differentiation. No potential intraepithelial precursor could be evaluated due to extensive ulceration. The lesion was diagnosed as a superficial portion of either an atypical fibroxanthoma (AFX) or of a pleomorphic dermal sarcoma (PDS). An extensive local re-resection with a 1-cm safety margin (Fig. 1C–D) was performed at a tertiary hospital centre three weeks after the shave biopsy. The final diagnosis of the AFX was made, since the tumour was microscopically contained within the cutis, with an epithelial collarette, and showed no evidence of subcutaneous or perineural invasion. At that time, the close proximity of the tumour tissue to the vascular clefts at the periphery (Fig. 1D) was not considered to be diagnostic of a vascular invasion. Sixteen months later, the patient presented at our hospital with bilateral arm weakness, dyspnoea, and orthopnoea. CT and MRI scans revealed an extensive osteolytic mass located in the third and fourth cervical vertebrae, with paravertebral, foraminal and epidural expansion as well as a pulmonary metastasis in the left lower lobe (Fig. 2A–B) with no other suspicious findings. Palliative surgical cervical decompression surgery with stabilisation was performed. Histologically, the diagnosis of bone metastasis of an undifferentiated malignant tumour was made (Fig. 2C–D). Extensive immunohistochemical analysis of the tumour tissue revealed in the analogy to the earlier skin lesion no specific line of differentiation, especially all epithelial (AE1/AE3, EMA), melanocytic (S100, SOX10, MART1, HMB45), myogenic (SMA, desmin, caldesmone) and endothelial (ERG, CD34) markers were negative. Additional reactions demonstrated a lack of the expression of PAX8, CD45, CD138, CD21, CD34, STAT6, MDM2, BRAF, ALK1, chromogranin, synaptophysin and CD56. The *SS18* gene FISH did not show rearrangement.

**Fig. 3:**
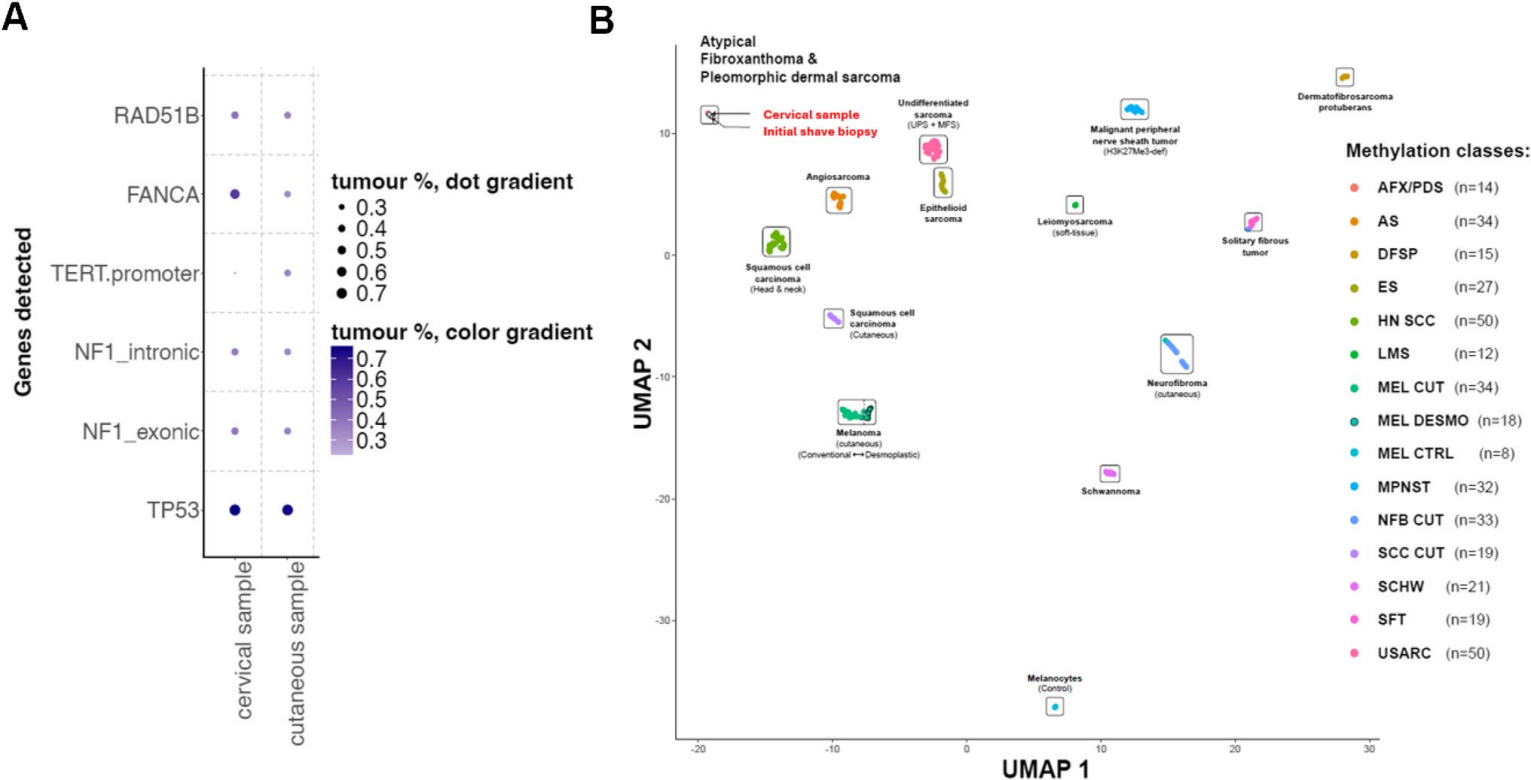
Summary of DNA sequencing and methylomic profile. (**A**) Dot plot showing somatic alterations detected by targeted panel sequencing in both tumours from the same patient. Genes are listed on the y-axis, and samples on the x-axis. Dot size and colour intensity represent the proportion of tumour cells harbouring the respective mutation, with darker and larger dots indicating higher allele frequencies. (**B**) UMAP representation of genome-wide DNA methylation profiles of a reference cohort of cutaneous and soft tissue tumours, including the two samples from this case (highlighted in red). Each point represents a sample, coloured by its methylation class as indicated in the legend (right). Both samples cluster tightly within the AFX/PDS methylation class (black)

As there was no other suspected primary tumour as the source of the hematogenic osseus and pulmonal metastatic spread, extensive molecular testing was performed to directly compare the skin tumour, which previously was diagnosed as an AFX, with the current metastatic tissue from the cervical spine (see below and Fig. 3). The cervical tumour mass progressed locally, and the patient died 10 weeks after the cervical operation. No autopsy was performed.

## Genetical analysis (DNA sequencing)

Samples of the skin tumour and the cervical spine metastasis were analyzed by parallel sequencing of DNA with the Oncomine™ Comprehensive Panel Version 3 (Thermo Fisher, USA) covering 135 genes (see supplementary information for a list of the genes covered). Cervical spine tumour sample was additionally analyzed using the FusionPlex™ Sarcoma Panel (Archer, USA) covering 137 genes and their fusion partners.

Sequencing analysis revealed high concordance in terms of single nucleotide variant (SNV) and Indels between both tumour samples (Fig. 3A). The following pathogenic or likely pathogenic mutations were found in both samples (skin and cervical lesions): *TP53*: p.R342Efs*3 at exon 10, *NF1*: p.R416* at exon 11, *NF1*: c.6820-1G > A at intron 45 (splice site), *TERT*: c.−124C > T at the promoter site, *FANCA*: c.792_792 + 1delinsAA at intron 8 (splice site), *RAD51B*: p.Q270* at exon 8.

In both samples, no gene amplification and no fusion transcript were identified.

Overall, DNA sequencing results are in favour of a close relation of a common origin of both tumour samples, respectively one being a metastasis and the other the primary tumour.

## Methylomic analysis

Infinium MethylationEPIC BeadChip microarray (HM850K; Illumina) was used to determine the genome-wide DNA methylation profile at single-base resolution covering over 850,000 CpG sites. Both tumour samples were analysed. Raw data files were pre-processed and normalized using the ChAMP Bioconductor package in R [13]. Normalisation was performed using BMIQ. Probes with single-nucleotide polymorphisms, cross reactive probes, and probes from X and Y chromosomes were also excluded. Each CpG site was assigned with a specific β value that is defined as the ratio of signal intensities between methylated (M) and unmethylated (U) probe that is β = M/(M + U). The β value ranges from 0 to 1, with 0 being unmethylated, and 1 fully methylated**.**

To compare the methylomic profile of our samples, we downloaded the IDAT files publicly available from reference dataset [10]. The samples labelled as “methylation class AFX/PDS” and “normal muscle tissue” were used as comparators. Using the R environment, unsupervised clustering was performed based on the 10,000 most variables CpG probes (Fig. 3B).

Methylomic analysis showed a highly similar clustering between the two samples confirming the manifestations of same tumour in both. In comparison with the reference samples, it revealed high concordant clustering with samples labelled as “methylation class AFX/PDS”. As shown in previous studies, DNA-methylation profiling cannot separate AFX from PDS [11]. Both samples did not cluster with reference cases of cutaneous conventional/desmoplastic malignant melanoma (Fig. 3B).

## Discussion and conclusion

We present a case of a well-documented skin tumour on the head of a 64-year-old man, initially diagnosed as atypical fibroxathoma (AFX) and accordingly adequately treated with an extensive local resection. However, fatal hematogenous metastatic disease developed within several months, raising questions about the accuracy of the initial diagnosis. As the immunohistochemistry remained inconclusive showing no signs of a specific line of differentiation, extensive molecular studies were performed to compare the genetic profiles of primary and metastatic tumours. Both the primary and metastatic tumours proved to be genetically related, with almost the same affected genes and the same types of the mutations in the *TP53*, *NF1*, *TERT* promoter, *FANCA* and *RAD51B* genes (Fig. 3A).

Mutations in the *TP53* und *TERT* promoter genes are common to AFX/PDS, squamous cell carcinomas, and malignant melanomas of sun exposed skin and are not sufficient to discriminate between these alternative diagnoses. However, the presence of the *NF1* gene mutations is typical of MM and, according to the literature is in fact the most common mutation in cases of dedifferentiated/undifferentiated malignant melanomas [7, 14, 15]. Interestingly however, the methylomic analysis demonstrated that the tumour clustered with reference cases of AFX/PDS and not with conventional or desmoplastic cutaneous malignant melanomas.

Routine histopathological diagnosis of an AFX remains challenging and is one of exclusion, being based on careful evaluation of the infiltration depth and extensive immunostains to rule out any signs of specific line of differentiation. According to the WHO definition, the diagnosis of PDS should be made not only when there is infiltration of the subcutaneous tissue or beyond, but also when there is vascular invasion [12]. In the current case, there was a very close relationship between the tumour tissue and to the vascular spaces retrospectively, so that the diagnosis of the PDS could have been considered. However, even if the vascular invasion had led to an original diagnosis of PDS, the extensive re-resection would still be considered adequate treatment in the non-metastatic setting. The clinical aggressiveness of the current tumour, with its extensive hematogenous metastatic disease is unusual for the entity from the AFX/PDS spectrum, as suggested by the methylomic analysis in comparison to reference cases and would better be consistent with an undifferentiated malignant melanoma as suggested the demonstration of the *NF1* gene mutations by NGS. Methylation signature of the cutaneous melanoma may be lost in cases of dedifferentiated melanomas as demonstrated by Hench et al. [9].

In conclusion, the routine histopathological diagnosis of tumours from the AFX/PDS spectrum, as we currently understand them, and undifferentiated malignant melanoma remains challenging. Molecular testing may help to correctly classify the cutaneous undifferentiated spindle cell and pleomorphic neoplasia. However, due to the relatively high and increasing incidence of such lesions in the ageing population, this costly and time-intensive analysis cannot currently be offered routinely for all cases.

## Supplementary Information

Below is the link to the electronic supplementary material.

Supplementary file1 (DOCX 31 KB)
