# Supplementary material for: Undifferentiated melanoma: a molecular study of a fatal metastatic “atypical fibroxanthoma (AFX)”
Source: Virchows Arch. 2025 Oct 11;487(5):1173–8. doi: 10.1007/s00428-025-04265-5 (PMC12647264; doi:10.1007/s00428-025-04265-5)
Supplement: Supplementary file 1 — Supplementary file1 (DOCX 31 KB) [file 428_2025_4265_MOESM1_ESM.docx]

Haefliger, S. et al: **Fatal metastatic skin tumour - undifferentiated melanoma or atypical fibroxanthoma?**

**Supplementary Material**

**Genes covered by the sequencing panel**

Oncomine™ Comprehensive Panel v3 (hotspots of 87 genes covered):

*AKT1,AKT2,AKT3,ALK,AR,ARAF,AXL,BRAF,BTK,CBL,CCND1,CDK4,CDK6,CHEK2,CSF1R,CTNNB1,DDR2,EGFR,ERBB2,ERBB3,ERBB4,ERCC2,ESR1,EZH2,FGFR1,FGFR2,FGFR3,FGFR4,FLT3,FOXL2,GATA2,GNA11,GNAQ,GNAS,H3F3A,HIST1H3B,HNF1A,HRAS,IDH1,IDH2,JAK1,JAK2,JAK3,KDR,KIT,KNSTRN,KRAS,MAGOH,MAP2K1,MAP2K2,MAP2K4,MAPK1,MAX,MDM4,MED12,MET,MTOR,MYC,MYCN,MYD88,NFE2L2,NRAS,NTRK1,NTRK2,NTRK3,PDGFRA,PDGFRB,PIK3CA,PIK3CB,PPP2R1A,PTPN11,RAC1,RAF1,RET,RHEB,RHOA,ROS1,SF3B1,SMAD4,SMO,SPOP,SRC,STAT3,TERT,TOP1,U2AF1,XPO1.*

Oncomine™ Comprehensive Panel v3 (48 genes fully covered):

*ARID1A,ATM,ATR,ATRX,BAP1,BRCA1,BRCA2,CDK12,CDKN1B,CDKN2A,CDKN2B,CHEK1,CREBBP,FANCA,FANCD2,FANCI,FBXW7,MLH1,MRE11A,MSH2,MSH6,NBN,NF1,NF2,NOTCH1,NOTCH2,NOTCH3,PALB2,PIK3R1,PMS2,POLE,PTCH1,PTEN,RAD50,RAD51,RAD51B,RAD51C,RAD51D,RB1,RNF43,SETD2,SLX4,SMARCA4,SMARCB1,STK11,TP53,TSC1,TSC2.*

Oncomine™ Comprehensive Panel v3: copy number variation, 47 genes covered:

*AKT1,AKT2,AKT3,ALK,AR,AXL,BRAF,CCND1,CCND2,CCND3,CCNE1,CDK2,CDK4,CDK6,EGFR,ERBB2,ESR1,FGF19,FGF3,FGFR1,FGFR2,FGFR3,FGFR4,FLT3,IGF1R,KIT,KRAS,MDM2,MDM4,MET,MYC,MYCL,MYCN,NTRK1,NTRK2,NTRK3,PDGFRA,PDGFRB,PIK3CA,PIK3CB,PPARG,RICTOR,TERT.*

Archer™ Custom V2 Panel, covering fusions involving following genes:

*ACVR2A, ALK, BCOR, BRAF, CAMTA1,CCNB3, CIC, CSF1, EGFR, EPC1, ERG, ETV1, EWSR1, FGF1, FGFR1, FGFR2, FGFR3, FGR,FOS, FOSB, FOXO1, FUS, GLI1, GRM1, HMGA2, IGF1R, JAZF1, MAML2, MEAF6, MET, MGEA5,MKL2, MSANTD3, MYBL1, MYB, NCOA2, NFATC2, NOTCH1, NRG1, NTRK1, NTRK2, NTRK3,NUTM1, PAX5, PDGFB, PHF1, PIK3CA, PLAG1, PPARG, PRKD1, RAF1, RET, ROS1, SRF, SS18,STAT6, TAF15, TCF12, TFE3, TFG, TMPRSS2, USP6, YWHAE.*

**Tissue selection, acid nucleic extraction**

For acid nucleic isolation, the area of interest was marked on H&E stained tissue slides and then macrodissected by scratching. The DNA was isolated using the Maxwell DNA purification kit according to the manufacturer’s protocol. The DNA concentration was quantified using the Qubit® dsDNA HS Assay Kit (ThermoFisher Scientific). The Ion AmpliSeq Library Kit V.2.0 (ThermoFisher Scientific) was used to prepare the libraries from 10-20 ng of DNA. The Ion Ampliseq HiFi Master Mix (ThermoFisher Scientific) was used to prepare the amplicons that were digested with FUPA reagent in order to remove primer-specific sequences and tagged with the IonCode Barcode Adapters. Finally, the amplified products were purified by performing a two-step cleanup using the Agencourt AMPure XP PCR purification system (Beckman Coulter, California, USA) at a bead to sample ratio of 1.15X and 1.0X, respectively. The Ion Library Equalizer Kit method was used to normalize the library concentration at ~100 pM. Finally, equal volumes of normalized DNA library were combined and amplified on Ion Sphere particles (ISP; ThermoFisher Scientific) by emulsion PCR using the Ion PI HiQ OT2 200 Kit (both ThermoFisher Scientific). Quality control was performed using the Ion Sphere Quality Control kit (ThermoFisher Scientific) to ensure that 10%–30% of template positive ISP were generated in the emulsion PCR. The template-positive Ion PI ISP were loaded on an Ion PI Chip and sequenced on an Ion S5^TM^ XLSequencer (ThermoFisher Scientific) with the Ion PI HiQ Sequencing 200 Kit (ThermoFisher Scientific) according to the manufacturer’s instructions.

**MATERIAL AND METHOD**

**Tissue selection and DNA extraction**

For DNA isolation, tissue blocks were cut to sections of 4 µm thickness, which were placed on a glass slide. Afterwards, the area of interest was marked by a pathologist using an H&E-stained tissue section as a guide. The selected area was macrodissected and DNA was isolated using the Maxwell RSC FFPE Plus DNA kit (Catalog number: AS1720) according to the manufacturer’s protocol. DNA was quantified by the Qubit dsDNA HS kit (Thermo Fisher Scientific, Cat.No. Q32854).

**Next-generation sequencing**

Both samples were analyzed by the Ion Torrent™ Oncomine Comprehensive™ Tumor Panel covering 135 genes with their exons (in brackets):

**Library Preparation**

The Ion AmpliSeq Library Kit 2.0 or Plus (ThermoFisher Scientific) was used to prepare the libraries from 10 ng of DNA using the Oncomine Solid Tumor Panel or the Ampliseq Cancer Hotspot Panel v2. First, the Ion Ampliseq HiFi Master Mix (ThermoFisher Scientific) was used to prepare the amplicons that were digested with FUPA reagent and tagged with barcode adapters. Next, the amplified products were purified using the Agencourt AMPure XP PCR purification system (Beckman Coulter, California, USA). The purified libraries were then diluted 1:1000 and quantified by qPCR using the Ion Universal Quantitation Kit (Thermo Fisher Scientific). The quantified stock libraries were then diluted to 50pM for downstream template preparation on the Ion Chef instrument (Thermo Fisher Scientific). NGS libraries were sequenced on an Ion S5™ instrument (Thermo Fisher Scientific) using semiconductor sequencing technology. Sequencing runs were planned on the Torrent Suite Software™ v5.6 or later and barcoded libraries were pooled and loaded on an Ion 530™ or 540™ chip using the Ion Chef™ instrument (Thermo Fisher Scientifc). The loaded chip was then inserted into the initialized Ion S5XL™ instrument and sequenced using 500 flows. Raw data was processed automatically on the Torrent Server™ and aligned to the reference hg19 genome. QC was performed manually for each sample aiming for the following metrics; on-target reads > 90%, read uniformity > 90%, and mean read depth > 2000. The sequencing data of the QC passing samples was then uploaded in BAM format to the Ion Reporter™ Analysis Server for variant calling and annotation.

Data analysis and statistical analysis

Variant detection was performed on the Ion Reporter™ Analysis Software v5.6 or later (ThermoFisher Scientific) using the appropriate workflow for either the Oncomine Solid Tumor Panel or Ampliseq Cancer Hotspot Panel v2. After variant calling, a filter chain was applied to remove polymorphisms based on cross-referencing with UCSC common SNPs, ExAC, 10000 Genomes, and 5000Exomes databases. Furthermore, remaining variants were filtered based on phred quality score > 100, allele read depth > 350, strand bias < 90%, minimal allelic frequency > 5%. Lastly, sequence variants were evaluated for their pathogenicity based on previous literature, databases (COSMIC, ClinVar, OncoKB, Varsome), and by using the open-access version from the Cancer Core Europe online portal.[13] Mutations were classified as pathogenic, likely pathogenic, variant of unknown significance (VUS), likely benign, and benign. Mutations classified as benign or likely benign were not reported. For statistical analysis, Fisher’s exact test was used. Statistical analyses were performed using R software package version 3.6.0 (www.r-project.org).
